# Identification of colorectal cancer patients with tumors carrying the *TP53 *mutation on the codon 72 proline allele that benefited most from 5-fluorouracil (5-FU) based postoperative chemotherapy

**DOI:** 10.1186/1471-2407-9-420

**Published:** 2009-12-02

**Authors:** Ten-i Godai, Tetsuji Suda, Nobuhiro Sugano, Kazuhito Tsuchida, Manabu Shiozawa, Hironobu Sekiguchi, Akiko Sekiyama, Mitsuyo Yoshihara, Shoichi Matsukuma, Yuji Sakuma, Eiju Tsuchiya, Yoichi Kameda, Makoto Akaike, Yohei Miyagi

**Affiliations:** 1 Department of Gastrointestinal Surgery, Kanagawa Cancer Center Hospital, 1-1-2 Nakao, Asahi-ku, Yokohama, Japan; 2 Molecular Pathology and Genetics Division, Kanagawa Cancer Center Research Institute, 1-1-2 Nakao, Asahi-ku, Yokohama, Japan; 3 Molecular Diagnostics, Kanagawa Cancer Center Hospital, 1-1-2 Nakao, Asahi-ku, Yokohama, Japan; 4 Department of Pathology, Kanagawa Cancer Center Hospital, 1-1-2 Nakao, Asahi-ku, Yokohama, Japan

## Abstract

**Background:**

Although postoperative chemotherapy is widely accepted as the standard modality for Dukes' stage C or earlier stage colorectal cancer (CRC) patients, biomarkers to predict those who may benefit from the therapy have not been identified. Previous *in vitro *and clinical investigations reported that CRC patients with wild-type p53 gene (*TP53)*-tumors benefit from 5-fluorouracil (5-FU) based chemotherapy, while those with mutated *TP53*-tumors do not. However, these studies evaluated the mutation-status of *TP53 *by immunohistochemistry with or without single-strand conformation polymorphism, and the mutation frequency was different from study to study. In addition, the polymorphic status at p53 codon 72, which results in arginine or proline residues (R72P) and is thought to influence the function of the protein significantly, was not examined.

**Methods:**

To evaluate the significance of the *TP53 *mutation as a molecular marker to predict the prognosis of CRC patients, especially those who received postoperative chemotherapy, we examined the mutation by direct sequencing from fresh CRC tumors and evaluated the R72P polymorphism of the mutated *TP53 *by a combined mutant allele- and polymorphic allele-specific polymerase chain reaction (PCR).

**Results:**

The *TP53 *mutation occurred in 147 (70%) of 211 Japanese CRC tumors. The mutation was observed in 93 (63%) tumors on the R72 allele and in 54 (37%) tumors on the P72 allele. Although the alterations to *TP53 *have no prognostic significance for CRC patients overall, we found that Dukes' stage C CRC patients who did not receive postoperative chemotherapy and carried the mutated *TP53*-R72 showed significantly longer survival times than those with the mutated *TP53*-P72 when evaluated by overall survival (*p = 0.012*).

**Conclusion:**

Using a combined mutant allele- and polymorphic allele-specific PCR, we defined the codon 72 polymorphic status of the *TP53 *mutated allele in Japanese CRC patients. We raised a possibility that Dukes' stage C colorectal cancer patients with tumors carrying *TP53 *mutation, especially the P72 allele, benefited from 5-FU based postoperative chemotherapy.

## Background

The tumor suppressor, p53, has a central role in stress responses that induce cell cycle arrest, senescence, apoptosis or DNA repair, and preserve genomic stability [1-6]. Therefore, dysfunction of the p53 pathway is a hallmark of neoplasms. Approximately half of all human tumors carry an alteration of the gene, *TP53 *[7]. Alterations of *TP53 *play a significant role in the progression of colorectal cancer (CRC) and may be a clinically useful molecular marker of prognosis or response to treatments such as chemotherapy, radiotherapy or combination of the two. *TP53 *is unique among tumor suppressor genes because its alteration not only results in loss-of-function of the product, but also generates a range of mutants demonstrating a gain-of-function phenotype. Li et al. and Blandino et al. reported that the expression of selected p53 mutants inhibited p53-independent apoptosis induced by the γ-irradiation and the anticancer reagents such as doxorubicin, cisplatin and etoposide [8,9]. Thereafter, in the last 10 years, the gain-of-function p53 mutants have been under intensive investigation because understanding the precise molecular mechanisms may provide information for personalized management and create promising therapeutic targets to benefit a large number of cancer patients [10,11].

More than 200 single nucleotide polymorphisms (SNPs) have been identified for *TP53*, 19 of which are exonic. Eleven of the exonic SNPs are non-synonymous resulting in an exchange of coding amino acid residues [10]. One of these SNPs results in either arginine (R) or proline (P) residues at codon 72 (R72P, rs1042522). A decade ago, this SNP gained attention when Storey et al. reported that the wild-type p53 with an arginine residue at codon 72 (hereafter p53-R72) is efficiently degraded by the E6 protein of the oncogenic type human papilloma viruses (HPV) and that p53-R72 homozygotes are at risk for HPV-associated uterine cervical cancer [12]. In contrast, later reports demonstrated that p53 with a proline residue at codon 72 (hereafter p53-P72) is associated with an increased risk in lung, esophagus, breast, urothelial and colorectal cancers [13-18]. However, other studies did not find any association with colorectal cancer [19,20]. The significance of the polymorphism R72P in association with the risk of cancer development or outcomes of cancer patients remains inconclusive, possibly due to differences in ethnic groups, genetic heterogeneity, sample number, cancer types, and treatment modalities in the each investigated cancer and population.

*TP53 *mutation in combination with the codon 72 polymorphic status has been examined extensively. Selected p53 mutants have been shown to gain the ability to bind to p73, a homologue of p53, and to inactivate the apoptosis pathway mediated by p73. Interestingly, this gain-of-function mutation is influenced by the p53 codon 72 polymorphism, and the mutated p53-R72 bind p73 more efficiently than the mutated p53-P72 [21]. As a result, the mutated p53-R72 show an enhanced dominant negative influence on the p73L/p73 pathway [22,23] and a significantly decreased response to cytotoxic chemotherapeutic reagents [24]. Although these *in vitro*-findings are partly supported by clinical investigations in several kinds of cancers [25-27], it is not yet addressed in CRC. Thus, in this study, we examined the polymorphism R72P of mutated *TP53 *in relation to the outcome of CRC patients who was received postoperative chemotherapy.

## Methods

### Patient population and tissue specimens

The study population consisted of 212 patients (122 men and 90 women; mean age 64.5 years) who had surgical resection of sporadic colorectal cancers during the period from 2002 to 2004 at Kanagawa Cancer Center Hospital. Cancer tissues were obtained from each case, and stored at -80°C until use. Patient outcome was followed either until their death or until July 31, 2008, and all the survival data of this study was restricted to 157 patients who passed at least 4 years after the operation. Demographic and clinicopathological characteristics of the patients included in the study are listed in Table 1. Pathologic stage was determined using the Dukes' staging system. Histopathologic types were described according to the World Health Organization Classification of Tumors [28]. Differentiation of adenocarcinomas was evaluated according to the TNM classification [29]. The number of lymph node metastases of advanced cancer patients was evaluated from pathological reports in at least 12 dissected lymph nodes. Ninety-four patients received postoperative chemotherapy. The 5-FU-based regimens included tegafur and uracil (UFT) following 5-FU: leucovorin (LV)/5-FU (RPMI regimen), 28 cases; LV/5-FU/CPT-11 (FOLFIRI/IFL), 11 cases; LV/UFT-based regimens, 55 cases. Twenty-six patients with Dukes' stage C did not agree to undergoing postoperative chemotherapy. The present study was approved by the institutional ethical review board at Kanagawa Cancer Center, and written informed consent to the study was obtained from all patients. Patients' follow-up data were obtained through a review of hospital and physician's records, by direct contact with the patient or through the social residential registration.

**Table 1:** Histological and clinical features of colorectal cancer patients (n = 212)

Gender (Male/Female)	122/90
Age (years +/- SD)	64.5 +/- 10.8
Primary tumor location	
Colon	114
Rectum	98
Dukes' stage, (%)	
A	42 (19.8)
B	61 (28.8)
C	75 (35.4)
D	34 (16.0)
Histopathological grade, (%)	
G1	44 (20.8)
G2	135 (63.7)
G3	33 (15.6)
Postoperative treatment	
Chemotherapy	94
No therapy	118

SD, standard deviation.

### Nucleic acid preparation and cDNA synthesis

Genomic DNA was isolated from tumor tissues using the QIAamp DNA Mini Kit (Qiagen KK, Tokyo, Japan) according to the manufacture's instructions. RNA was extracted with the TRIZOL^® ^Reagent (Invitrogen, Carlsbad, CA), following the manufacture's instructions. First-strand cDNA was synthesized with an oligo(dT)_12-18 _primer and the SuperScript^® ^First-Strand Synthesis System for RT-PCR (Invitrogen).

### *TP53 *mutation analysis

Mutational analysis of *TP53 *was performed by direct sequencing. Briefly, a 1.64 kb fragment of *TP53 *(corresponding to exons 5-8) was amplified from genomic DNA extracted from each tumor by polymerase chain reaction (PCR). The PCR products were purified and directly sequenced using GenomeLab™ DTCS Quick Start Kit and the CEQ™ 2000XL DNA Analysis System (Beckman Coulter, Inc., Fullerton, CA). The obtained nucleotide sequences were compared with the *TP53 *reference sequence (GenBank accession number X54156). The primers used to amplify exons 5-8 of *TP53 *are available on request.

### Determination of the *TP53 *codon 72 polymorphic status of tumors

For the determination of polymorphism at codon 72 of *TP53*, an allele-specific PCR assay was used as described previously [12,30] with minor modifications. The precise information for PCR conditions is provided in Additional file 1.

For the *TP53 *mutated tumors with arginine/proline heterogeneous polymorphic status at codon 72, a combined method of the polymorphic allele-specific [31] and the mutant allele-specific [32] PCR was performed to determine whether the *TP53 *mutation occurred on the arginine allele or on the proline allele. Briefly, the method was designed to place the polymorphic nucleotides at the 3'-end of the forward primers and the mutated sites at the 3'-end of the reverse primers. The nucleotide sequences of the two polymorphic allele specific primers were 5'-gaggctgctccccg-3' for the arginine allele and 5'-gaggctgctccccc-3' for the proline allele. The nucleotide sequences of the reverse primers, specific to each mutation site, are listed in Additional file 2. The precise information for PCR conditions is also provided in Additional file 1.

### Statistical Methods

The relationship between the codon 72 polymorphic status of the mutated allele of *TP53 *and individual clinicopathological variables were assessed using chi-square tests. Overall survival curves were generated by Kaplan-Meier analysis, and the log rank test was used to compare between survival curves. Differences were considered significant when a P value < 0.05 was obtained. All the statistic analyses were performed by SPSS version 15 for Windows (SPSS Inc. Chicago, IL).

## Results

### *TP53 *codon 72 polymorphic status of the tumors

**Figure 1:**
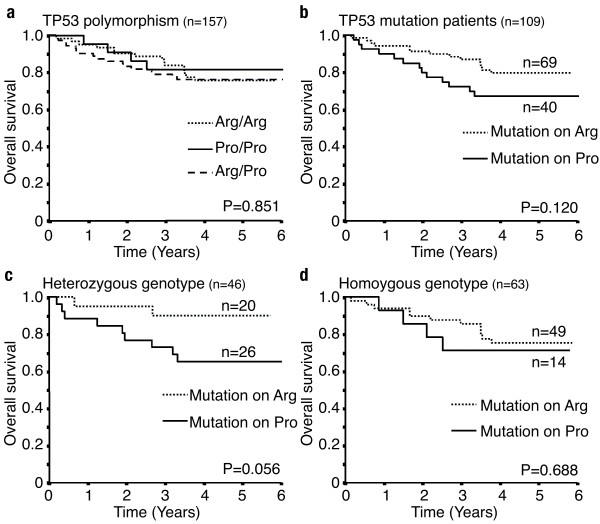
**Effect of *TP53 *codon 72 genotype on overall survival in colorectal cancer patients**. (a): The association between the *TP53 *codon 72 genotype and overall survival. (b): Overall survival with *TP53 *mutation on the Arg allele and Pro allele (n = 109. P = 0.120). (c): Overall survival in heterozygous patients with *TP53 *mutation (n = 46, P = 0.056). (d): Overall survival in homo- and hemizygous genotype patients with *TP53 *mutation (n = 63, P = 0.688).

We analyzed the polymorphic status of the *TP53 *codon 72 by allele-specific PCR analysis. As shown in Table 2, the allele frequencies were determined as 42% (89 tumors) for the arginine allele, 45% (94 tumors) for the heterozygous arginine/proline status, and 13% (28 tumors) for the proline allele, respectively. There was no significant difference in the overall patient survival among the three codon 72 status groups as assessed by the Kaplan-Meier analysis (Figure 1a).

**Table 2:** Genotype frequencies at codon 72 of *TP53 *in colorectal cancer patients (n = 211)

	Arg	Arg/Pro	Pro	P value
Cases, n (%)	89 (42.2)	94 (44.5)	28 (13.3)	
Age (years +/- SD)	64.7 +/- 10.8	64.8 +/- 11.2	63.2 +/- 10.2	
Size (cm), n (%)				
<5	48 (42.5)	52 (46.0)	13 (11.5)	
≥ 5	41 (41.8)	42 (42.9)	15 (15.3)	0.707
p53 mutations, n (%)				
wt	22 (34.4)	32 (50.0)	10 (15.6)	
mt	67 (45.6)	62 (42.2)	18 (12.2)	0.313
Mutations by codon 72 status, n				
Mutation on Arg	67	26	-	
Mutation on Pro	-	36	18	
Dukes' stage, n (%)				
A	17 (40.5)	21 (50.0)	4 (9.5)	
B	25 (41.7)	27 (45.0)	8 (13.3)	
C	31 (41.3)	30 (40.0)	14 (18.7)	
D	16 (47.1)	16 (47.1)	2 (5.9)	0.622

### *TP53 *mutations of the tumors

*TP53 *mutation occurred in 148 (70%) of the tumors. One patient with tumors heterozygous for the codon 72 polymorphism had two different *TP53 *mutations on each allele. Therefore, we removed this case and used 211 patients for the following analyses (Table 2). Individual mutations in detail and the codon 72 allelism are shown in Additional file 3, 4. Briefly, we found 107 missense (73%), 8 frameshift (5%), 9 splice (6%), 17 nonsense (12%), 2 missense+nonsense (1%) and 4 other mutations. Silent mutation was considered as wild-type in the present study for analyses. Eleven non-synonymous polymorphisms, including the codon 72 (R72P), are now recognized in *TP53*. We evaluated 5 polymorphic sites except for R72P located in exons 5-8, including V217 M, R267W, P278A, R290H and N311S [10], respectively. In the present study, all the examined sequences corresponding to the above polymorphic sites matched completely to the *TP53 *reference sequence used, and no exonic non-synonymous polymorphism other than R72P was observed.

### *TP53 *codon 72 status with or without mutations and patient outcome

We determined *TP53 *mutated allele for the 62 tumors heterozygous for the codon 72 polymorphic status. Twenty-six *TP53 *mutations occurred on the arginine allele and 36 mutations occurred on the proline allele (Table 2). In total, *TP53 *mutation occurred on the arginine allele in 93 tumors (44%) and on the proline allele in 54 tumors (26%) among the 211 informative colorectal cancer samples (Table 3). The lymph node metastasis was significantly increased with the *TP53 *mutation (p < 0.05, chi-square test; Table 3). However, the background factors of age, sex, number of lymph node metastasis, tumor size, metastasis, venous involvement and lymphatic involvement were not statistically different between the tumors consisting of the mutated *TP53*-R72 and the tumors with the mutated *TP53*-P72 (data not shown). Likewise, there were no significant differences in the overall survival between patients with the mutated *TP53-*R72 and with the mutated *TP53-*P72 (Figure 1b). In the present analysis, we were not able to distinguish tumors with mutated *TP53-*R72 together with wild-type *TP53-*R72 from tumors lacking wild-type *TP53*-R72 by LOH. Concerning *TP53*-P72, the findings were the same. Therefore, we selected tumors with mutated *TP53 *under heterozygous codon 72 status, a definitive retention of the other wild-type allele, and analyzed the overall patient survival. There was a tendency that the mutated *TP53*-P72 showed a poorer prognosis than that of the mutated *TP53-*R72, but the difference was not significant (Figure 1c). There was no significant difference in the overall patient survival between patients with the mutated *TP53*-R72 and patients with the mutated *TP53*-P72 under homozygous codon 72 status, which includes both LOH tumors and tumors retaining the homologous wild-type allele. There was also no significant difference in the overall patient survival between the tumors with the mutated *TP53*-R72 and the mutated *TP53*-P72 (Figure 1d).

**Table 3:** *TP53 *mutation and allelism (n = 211)

	No mutation	Mutation on Arg	Mutation on Pro	P value
Case, n (%)	64 (30.3)	93 (44.1)	54 (25.6)	
Primary tumor location, n (%)				
Colon	35 (31.0)	51 (45.1)	27 (23.9)	
Rectum	29 (29.6)	42 (42.9)	27 (27.6)	0.832
Site, n (%)				
Proximal	18 (36.7)	17 (34.7)	14 (28.6)	
Distal	17 (26.6)	34 (53.1)	13 (20.3)	0.149
Histopathological grade, n (%)				
G1+G2	50 (28.1)	79 (44.4)	49 (27.5)	
G3	14 (42.4)	14 (42.4)	5 (15.2)	0.167
Lymph node metastasis, n (%)				
N0	43 (40.6)	39 (36.8)	24 (22.6)	
N1	16 (19.0)	45 (53.6)	23 (27.4)	
N2	5 (23.8)	9 (42.9)	7 (33.3)	0.022*
Dukes' stage, n (%)				
A	19 (45.2)	15 (35.7)	8 (19.0)	
B	23 (38.3)	21 (35.0)	16 (26.7)	
C	12 (16.0)	42 (56.0)	21 (28.0)	
D	10 (29.4)	15 (44.1)	9 (26.5)	0.024*

* The statistical difference is shown by the chi-square test.

### Significance of the codon 72 status on the clinicopathological factors

The CRC specimens were stratified by the location (colon or rectum), the histological subclasses and the Dukes' stage (Table 2). The frequency of mutation that occurred on the *TP53*-R72 or -P72 was not statistically different in tumor location or histological subclass (Table 3).

The *TP53 *mutation rate showed a tendency to increase with the Dukes' stage progression to stage C. The *TP53 *mutation was 55% (23 out of 42) in Dukes' stage A patients and 84% (63 out of 75) in stage C patients, and this difference was statistically significant (p < 0.05, chi-square test; Table 3). However, frequency of the mutations that occurred on the *TP53*-R72 or -P72 and the Dukes' stage showed no significant relevance (p > 0.05, chi-square test). In addition, the overall survival of the patients was not different between the mutated alleles and the Dukes' stages (data not shown).

### Significance of the codon 72 status of the mutated *TP53 *on the patient outcome after postoperative chemotherapy

**Figure 2:**
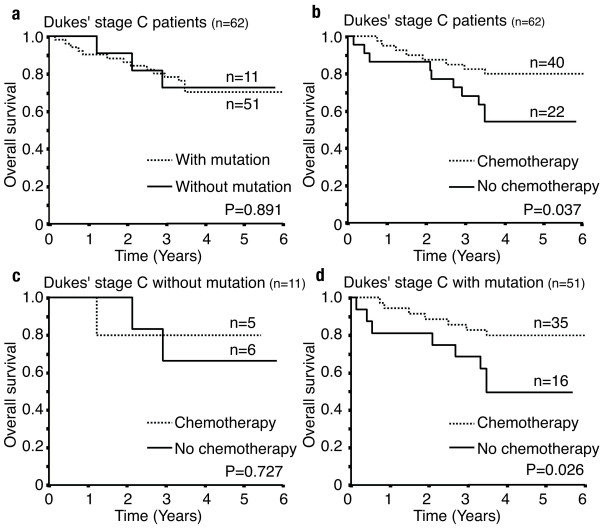
**Effect of chemotherapy and the *TP53 *mutation in colorectal cancer patients of Dukes' stage C**. (a): Overall survival of Dukes' stage C patients with the *TP53 *mutation and without the mutation (n = 62, P = 0.891). (b): Overall survival of patients with surgical treatment alone and with postoperative chemotherapy (n = 62, P = 0.037). Patients at Dukes' stage C with the *TP53 *mutation did not show significant sensitivity to chemotherapy (c) (n = 11, P = 0.727), but patients with the *TP53 *mutation show a significant effect of chemotherapy (d)(n = 51, P = 0.026).

**Figure 3:**
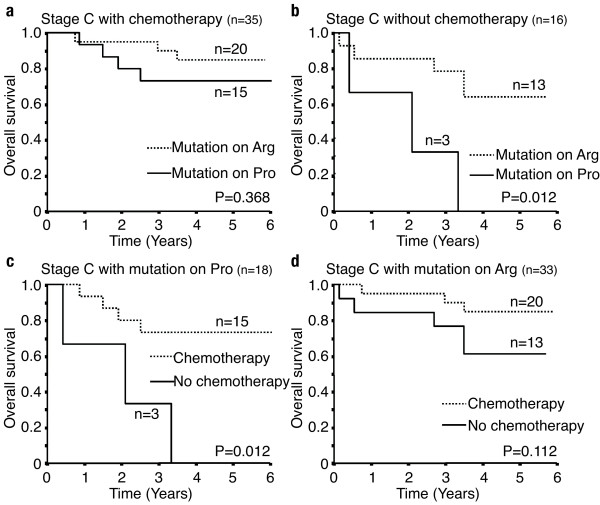
**Effect of chemotherapy and the *TP53 *mutation on codon 72 polymorphic allele in Dukes' stage C patients**. (a): Overall survival of a Dukes' stage C and chemotherapy patient with the *TP53 *mutation of the Arg allele and Pro allele (n = 35, P = 0.368). (b): Overall survival of patients without chemotherapy and with *TP53 *mutation of the Arg and Pro allele (n = 16, P = 0.012). Dukes' stage C patients with *TP53 *mutation on the Pro allele show a significant effect of chemotherapy (c) (n = 18, P = 0.012), but the patients with mutation on the Arg allele did not reach statistical significance (d)(n = 33, P = 0.112).

Fifty-four patients with *TP53 *mutation received postoperative chemotherapy, and there was no significant difference in the overall patient survival between the two codon 72 polymorphic status groups of the mutated alleles (n = 32 for R72 and n = 22 for P72) (p = 0.475; data not shown). Then, we focused on the stage C patients. *TP53 *mutation had no relation to the overall patient survival (Figure 2a). And there was no significant difference in the overall survival after postoperative chemotherapy among the patients without *TP53 *mutation (Figure 2c). However, all patients, or the patients with *TP53 *mutation, who received postoperative chemotherapy, showed a significantly better survival than the patients without chemotherapy (Figure 2b, d). Focusing on the codon 72 polymorphic status of the mutated *TP53*, survival after chemotherapy was not different between the *TP53*-R72 and *TP53*-P72 (Figure 3a). By contrast, as concerns the 16 patients with mutated *TP53*, who did not receive postoperative chemotherapy, the patients with the mutated *TP53*-R72 (n = 13) showed significantly longer survival than those with the mutated *TP53*-P72 (n = 3) (p = 0.012; Figure 3b). Among the patients with the mutated *TP53*-P72, the patients who received postoperative chemotherapy (n = 15) showed significantly better prognosis than those without chemotherapy (n = 3) (p = 0.012; Figure 3c). Although the tendency for a better prognosis was demonstrated by similar analysis, a statistically significant difference was not observed in the patients with the mutated *TP53*-R72 (p = 0.112; Figure 3d).

### *TP53 *mutations showing effective p73 inhibition *in vitro *and the patient outcome

We tested whether patients with 18 *TP53 *mutated allelotypes [24] demonstrate a poor prognosis or resistance to postoperative chemotherapy. Twenty-seven patients (5 in Dukes' stage A, 6 in stage B, 11 in stage C, and 5 in stage D) had such tumors. However, there were no statistical differences of survival when compared with the others overall, in each Dukes' stage, in patients that received chemotherapy or in patients without chemotherapy (data not shown).

## Discussion

Although postoperative adjuvant therapy is accepted widely as the standard modality for stage III, Dukes' stage C or earlier stage CRC patients, Tang et al. reported that one-fourth of stage III patients and two-thirds of stage II patients received no such therapy [33]. Those patients tended to be older and to have less advanced tumors when compared with those who received postoperative adjuvant therapy. Therefore, predicting the patients who benefit from postoperative adjuvant therapy is important to selectively recommend the therapy and to improve the prognosis.

Although alterations of *TP53 *appear to have little- or no-prognostic significance for CRC patients treated with surgery alone [34], several clinical studies have demonstrated that CRC patients with wild-type *TP53*-tumors gain a survival benefit from 5-FU based postoperative chemotherapy, but those with *TP53*-mutated tumors do not [35-37]. Westra et al. also showed the same results from stage III colon cancer patients [35]. These studies were conducted in Australia, Europe and the United States. A large cohort, "the *TP53*-CRC-international collaborative study", which is a meta-analysis study analyzing more than 3,500 CRC patients from 17 countries, also demonstrated that Dukes' C tumors with wild-type *TP53 *showed a significantly better survival when treated with postoperative adjuvant chemotherapy [38]. This extensive meta-analysis, however, involved 2 Japanese studies with 103 CRC patients, corresponding to only 2.9% of all cases evaluated, and the *TP53 *mutation frequency ranged widely from 31% to 84%. In the present study, we included Japanese CRC patients from a single institute, and observed *TP53 *mutation in 70% of colorectal cancers. The Dukes' stage C patients who received postoperative chemotherapy showed a better overall survival than those who did not. This is consistent with previous investigations. However, the patients that benefited from the 5-FU based postoperative chemotherapy were not the patients with tumors containing wild-type *TP53*, but those with tumors containing *TP53 *mutation. Although Bunz et al. demonstrated *in vitro *that the apoptosis-inducing effect of 5-FU was almost abrogated when they deleted *TP53 *in the wild-type *TP53 *carrying human colon cancer cell, HCT116 [39], this situation is quite different from the actual clinical setting in which p53 function was knocked out not by homozygous deletion, but by missense mutation. Actually, we found that 72% of the *TP53 *alterations were missense mutation, which may result in gain-of-function oncogenic properties. Our results suggest that the oncogenic property of mutated p53 is involved significantly in the malignant phenotype of these tumors, and that the 5-FU based postoperative chemotherapy is effective.

Longley et al. reported that 5-FU increased Fas protein expression in the wild-type *TP53 *colon cancer HCT116 cells, but not in the *TP53*-null cells or *TP53-*mutated colon cancer H630 cells [40]. Our results showed that the tumors carrying the mutation on *TP53*-P72 benefited most from the 5-FU based postoperative chemotherapy seems contradictory to the results of those reports. However, it is unclear what *TP53 *mutation occurred in H630 and how the oncogenic property of p53 gain-of-function mutation in combination with R72P status influences the apoptosis system. Therefore, there may be unknown pathways of apoptosis that function preferentially in colorectal cancer cells with the mutated *TP53*-P72 in response to 5-FU based postoperative chemotherapy.

Predicting which patients will benefit from the postoperative adjuvant therapy is important to improve CRC prognosis, and the prediction may be different when different adjuvant therapies are performed. In the present study, we identified CRC patients with tumors carrying *TP53 *mutation, especially on the codon 72 proline allele, as those patients that benefited most from 5-FU based postoperative chemotherapy. This result seems to be contradictory to several previous clinical reports or *in vitro *studies, but, we evaluated *TP53 *mutation by direct sequencing on fresh tumor samples and the precise discrimination of polymorphic status of the codon 72 were different from previous studies. The ethnic background of the investigated population may also be responsible for the differences.

## Conclusion

In the present study, we identified the codon 72 polymorphic status of the *TP53 *mutated allele in CRC patients. We found that Dukes' stage C CRC patients with the mutated *TP53*-R72 who did not receive postoperative chemotherapy showed significantly longer survival times than those with the mutated *TP53*-P72. Our results raised a possibility that Dukes' stage C CRC patients with tumors carrying *TP53 *mutation, especially the P72 allele, benefited most from 5-FU based postoperative chemotherapy, but we need to collect more CRC cases to form definite conclusions.

## Competing interests

The authors declare that they have no competing interests.

## Authors' contributions

TG and TS performed the statistical analysis and drafted the manuscript. TG, TS, HS, AS and MY carried out sequencing and PCR analysis. TG, NS, KT, MS and MA collected and analyzed samples from colorectal cancer patients. YK performed pathological analysis and revised the manuscript. SM, YS, ET reviewed all data, and contributed to the preparation of the manuscript. YM directed the overall project, and participated in the editing of the final manuscript. All authors read and approved the final manuscript.

## Pre-publication history

The pre-publication history for this paper can be accessed here:

http://www.biomedcentral.com/1471-2407/9/420/prepub

## Supplementary Material

Additional file 1 **Determination of the *TP53 *codon 72 polymorphic status of tumors**. The precise information for PCR conditions. Click here for file

Additional file 2 **Reverse primers used for mutant-allele-specific amplification**. The nucleotide sequences of the reverse primers, specific to each mutation site. Click here for file

Additional file 3 **Individual mutations in detail and the codon 72 allelism**. Precise information for the *TP53 *mutation found in each case was provided. Click here for file

Additional file 4 **Location and frequency of p53 missense mutation in the p53 protein**. The positions of the mutated amino acid of p53 and the number of mutations in exons 5-8 are shown by the black bar. One hundred and five tumors corresponded to transitions (58 G:A and 52 C:T) and 22 transversions (4 A:C, 1 A:T, 14 G:T, and 3 G:C) were identified. Four mutations identified in the present study (c.560-19_560-9del11, c.571-586del16, c.625_628delAGAA and c.673-16_673-4del13) have not been described in the 4 major databases for *TP53 *mutation: the IARC *TP53 *mutation database http://www-p53.iarc.fr/ [Petitjean A *et al*., *Hum Mutat *2007], the database of germline p53 mutations http://www.lf2.cuni.cz/projects/germline_mut_p53.htm, the *TP53 *knowledgebase http://p53.bii.a-star.edu.sg/index.php, and the *TP53 *web site http://p53.free.fr/. Click here for file
