# Supplementary material for: Identification of colorectal cancer patients with tumors carrying the TP53 mutation on the codon 72 proline allele that benefited most from 5-fluorouracil (5-FU) based postoperative chemotherapy
Source: BMC Cancer. 2009 Dec 2;9:420. doi: 10.1186/1471-2407-9-420 (PMC2796677; doi:10.1186/1471-2407-9-420)
Supplement: Additional file 1 — Determination of the TP53 codon 72 polymorphic status of tumors. The precise information for PCR conditions. [file 1471-2407-9-420-S1.DOC]

**Additional file 1**

# Determination of the *TP53* codon 72 polymorphic status of tumors

For the determination of polymorphism at codon 72 of *TP53*, an allele-specific PCR assay was used as described previously [12][30] with minor modifications. Briefly, the allele-specific PCR was carried out in a total volume of 25 µl, consisting of 50 ng of DNA, 1xPCR buffer without Mg^++^ (supplied by the manufacturer), 0.8 mM (to amplify the arginine type allele) or 1 mM (to amplify the proline type allele) MgCl_2_, 120 µM (arginine type) or 150 µM (proline type) of dNTPs, 100 nM (arginine type) or 200 nM (proline type) of each PCR primer and 1.25 units of Platinum® Taq DNA polymerase (Invitrogen). PCR amplification was performed with an initial denaturation step at 94 ˚C for 100 sec, followed by 35 cycles of denaturation at 94 ˚C for 20 sec, annealing at 60 ˚C for 20 sec, and extension at 72 ˚C for 20 sec, and a final extension step at 72 ˚C for 5 min. The sequences of primers were as described previously [30]. The PCR products for the respective polymorphic status were visualized in a 2% agarose gel by electrophoresis.

For the *TP53* mutated tumors with arginine/proline heterogeneous polymorphic status at codon 72, a combined method of the polymorphic allele-specific [31] and the mutant allele-specific [32] PCR was performed to determine whether the *TP53* mutation occurred on the arginine allele or on the proline allele. Briefly, the method was designed to place the polymorphic nucleotides at the 3’-end of the forward primers and the mutated sites at the 3’-end of the reverse primers. The nucleotide sequences of the two polymorphic allele specific primers were 5’-gaggctgctccccg-3’ for the arginine allele and 5’-gaggctgctccccc-3’ for the proline allele. The nucleotide sequences of the reverse primers, specific to each mutation site, are listed in Additional file 2. Each PCR reaction contained 25 ng of cDNA, 1xPCR buffer without Mg^++^ (supplied by the manufacturer), 200 nM of each primer, 1.5 to 3 mM MgCl_2_ and 200 µM of dNTPs. The PCR conditions and concentration of MgCl_2_ were determined for each primer set (the conditions are available on request). Amplified products were separated on a 2% agarose gel and visualized by ethidium bromide staining. The codon 72 status of the *TP53* mutated allele was determined by the polymorphic primer that gave the amplified PCR product.
